# Synthesis and Heavy-Metal Sorption Studies of *N*,*N*-Dimethylacrylamide-Based Hydrogels

**DOI:** 10.3390/polym13183084

**Published:** 2021-09-13

**Authors:** Ayatzhan Akhmetzhan, Nurgeldi Abeu, Sotirios Nik. Longinos, Ayezkhan Tashenov, Nurbala Myrzakhmetova, Nurgul Amangeldi, Zhanar Kuanyshova, Zhanar Ospanova, Zhexenbek Toktarbay

**Affiliations:** 1 Faculty of Natural Sciecnes, L.N. Gumilyov Eurasian National University, Kazhymukan Street 5, Astana 010000, Kazakhstan; aiatzhan@gmail.com (A.A.); tashenov_ak@enu.kz (A.T.); 2 Department of Analytical, Colloidal Chemistry and Technology of Rare Elements, Faculty of Chemistry and Chemical Technologies, Al-Farabi Kazakh National University, Al-Farabi Av. 71, Almaty 050040, Kazakhstan; nurgeldi-abeu@mail.ru (N.A.); Zhanar.Ospanova@kaznu.kz (Z.O.); 3 Department of Petroleum Engineering, Nazarbayev University, Kabanbaybatyr Av. 53, Nur-Sultan 010000, Kazakhstan; s.n.longinos@gmail.com; 4 Department of Chemistry, Faculty of Natural Science, Kazakh National Woman’s Teacher Training University, Aitekebi Street 99, Almaty 050000, Kazakhstan; nmyrzahmetova64@gmail.com (N.M.); nurgul.amang1987@gmail.com (N.A.); zhanarkuanysheva.111@gmail.com (Z.K.)

**Keywords:** hydrogel, DMAA, AMPS, swelling ratio, crosslinker, metal-ion adsorption

## Abstract

In this work, a hydrogel system was produced via radical polymerization of *N*,*N*-dimethylacrylamide and 2-acrylamido-2-methylpropanesulfonic acid in the presence of *N*,*N*-methylene-bis-acrylamide as a crosslinker and ammonium persulfate as an initiator. Parameters that impact the conversion of copolymerization (such as initial concentration of monomers, temperature, initiator dose, and time) were studied. The swelling degree of the hydrogel was investigated with the addition of a crosslinker and initiator at different pH levels. A hydrogel with high conversion and high swelling degree was selected to investigate their ability for adsorption of Pb(II) ions from solutions. Adsorption behavior of Pb(II) ions in a hydrogel was examined as a function of reaction time and concentration of lead ions from a solution of Pb(II) ions.

## 1. Introduction

In recent years, hydrogels have received broad interest due their unique properties, such as elasticity and swelling. Hydrogels produced from crosslinked polymers have been widely used in agriculture [1], gene delivery [2], drug delivery [3,4,5], thermo sensitive materials [6], pharmaceutical [7], food industry [8], diagnostics [9], wound dressing [10], contact lenses [11], etc.

Among them, poly(acrylamide)-based hydrogel has been applied in many areas including dye removal [12], heavy-metal removal [13], film making [14], tissue engineering [15], smart polymers [16], and linear polymers [17]. An *N*,*N*-dimethylacrylamide (DMAA) is an N-substituted dimethyl acrylamide monomer that produces such hydrogels as crosslinked by various crosslinkers.

Zaborina et al. prepared poly(DMAA) homopolymers in frozen media using red-ox initiator and a small number of crosslinkers, which resulted in crosslinked high-swelling cryogels [18]. In combination with other monomers, DMAA is applied to produce a wide variety of materials, which include the application of hydrogel to protect seeds in soils and enhance their biocompatibility in medical fields [19]. As well as these, DMAA can be used to form intermolecular complexes with polyelectrolytes, such as polyacrylic acid, which is applied as a smart material with changes of pH [20]. 

In copolymerization with *N*,*N*-dimethylacrylamide (DMAA), 2-acrylamido-2-methylpropanesulfonic acid (AMPS) was used to increase the water retention properties of hydrogel, since AMPS has the capability of large water adsorption [21,22]. The sulfonate group dissociates in all pH ranges [23,24]. Therefore, AMPS hydrogels exhibit pH-independent swelling behavior, which gives them applications across a wide variety of fields [25,26,27]. The negatively charged sulfate ions of AMPS usually pair with metal ions [28,29]. 

In many studies, copolymers of DMAA and AMPS with acrylic acids and amides were studied. However, there are too few or no studies that have investigated the kinetics of synthesis and swelling degree depending on different parameters such as crosslinking degree and monomer concentration, etc. In the present study, we intended to investigate the formation of a hydrogel consisting of a copolymer of DMAA and AMPS with *N*,*N*′-methylenebis(acrylamide) as crosslinker, using a change of pH, concentration of crosslinking agent, initiator, monomer, and temperature, and their swelling behavior. The aim of the study was to find the optimum condition for the synthesis of such hydrogels. Finally, we tested the synthesized hydrogel for metal-ion absorbency. 

## 2. Materials and Methods

### 2.1. Materials

Monomers: *N*,*N*-dimethylacrylamide (DMAA) (code: 274135) and 2-acrylamido-2-propane sulfonic acid (AMPS) (code: 282731), crosslinking agent: *N*,*N*′-methylenebis(acrylamide) (code: 146072), and lead (II) nitrate (code: 228621). The above chemicals were purchased from Sigma–Aldrich (St. Louis, Missouri, USA). Ammonium persulfate (NH_4_)_2_S_2_O_8_ (purity 99.7 wt.%) (Initiator) was received from “LaborPharma” Ltd. (Almaty, Kazakhstan). All chemicals were used as obtained without any further purification. Acetone (purity 99.9 wt.%) and all the other organic compounds were bought from “Labchimprom” Ltd. (Almaty, Kazakhstan). Argon (purity 99.995 wt.%) was ordered from “Ikhsan” Ltd. (Almaty, Kazakhstan). In all experiments, distilled water was used with electrical conductivity of 2.4 mS/cm at 20 °C.

### 2.2. Copolymerization

In the presence of (NH_4_)_2_S_2_O_8_ initiator, polymerization reaction of Na-AMPS with DMAA was performed at different molar ratios of monomers. The initiator concentration used was from 0.05 to 0.1 wt.% of total mass of the monomers. The crosslinker concentration was in the rage of 1.5–8.5 wt.% of total weight of the monomers. The ampoule containing the monomers was sealed after purging with argon for 20 min and placed in a water bath at controlled temperatures. After the required time, the hydrogels (copolymers) were washed with fresh acetone to eliminate the unreacted monomers. All the copolymers were then dried to constant weight under vacuum at 40 °C. 

### 2.3. Conversion of Polymerization

Percent conversions of polymerization were calculated by polymer weight yield percentage. After polymers were eliminated from unreacted monomers by washing fresh acetone several times, then polymers were dried to constant weight. Dried polymer weights were divided by the starting monomer weight and multiplied by 100%. 

### 2.4. Swelling Measurements

The hydrogels were soaked in distilled water at 25 °C for 3 days to reach a swelling equilibrium. The hydrogel was examined by weighing its mass after the swollen hydrogel was further filtered by paper and cleaned with water. The swelling ratio (%Sr) was calculated using the following equation.

(1) $$\%\mathrm{Sr}=\frac{M_{s}-M_{d}}{M_{d}}\;\cdot 100\%$$

here, M_s_ and M_d_ are the mass of initial and swollen hydrogel, respectively. The influence of pH on the swelling degree was tested at the different pH levels. pH of the solution was adjusted by NaOH and HCl solutions.

Likewise, swelling of the hydrogel depending on the crosslinker concentration was investigated as a concentration of crosslinkers ranging from 1.5 to 8.5 wt.% of total weight of the monomers.

### 2.5. Metal-Ion Absorbency Studies 

The prepared hydrogel was also used to investigate adsorption of Pb(II) metal ion from the metal-ion solutions. The Pb(II) ion solution was prepared by dissolving lead nitrate in deionized water, at 298 K. In brief, 100 mg of polymer (hydrogel) sample was placed in 15.0 mL solutions of metal ion with different concentrations (30, 50, and 70 mg/L). At certain times, the hydrogel was taken out and the supernatants were further examined to find absorbed ions. The concentration of the metal ion was determined on a UV-7504 spectrophotometer using its standard reagents. 

The amount of adsorbed lead ions at any time t (Q_t_, mg/g) and at equilibrium (Q_e_, mg/g) was determined using the following equations:

(2) $$Q_{t}=\frac{(C_{0}-C_{t})*V}{m}$$

(3) $$Q_{e}=\frac{(C_{0}-C_{e})*V}{m}$$

where C_0_, C_t_, and C_e_ (mg/L) represent initial concentration, concentration at time t, and equilibrium of lead ions respectively; V (L) is the volume of lead ion solution, and m (g) is the mass of the dry hydrogel [30].

### 2.6. Data Presentation and Statistical Analysis 

Two of the experiments were duplicated (Table 1 and Table 2), each time finding very similar results (very small standard error values). 

For example, in Figure 5, for concentrations of 0.45% and 0.85% (their selection was random) we calculated the standard errors and the results are written below: 

For example, for concentration 0.45% for 1 h $SE_{x}=\frac{SE}{\sqrt{n}}$, n is number of experiments, which in this instance is always 2. 

Data were expressed as mean ± standard deviation. The values *p* < 0.05 were considered statistically significant. Statistical analysis was done using MS Excel 2010 software version 14.0 (Washington, USA). Pearson’s correlation was used to analyze the association between all studied parameters.

## 3. Results and Discussion

In this research, the effect of different parameters, including the concentration of monomers, initiator, and crosslinkers on polymer conversion, was investigated. 

**Figure 1:**
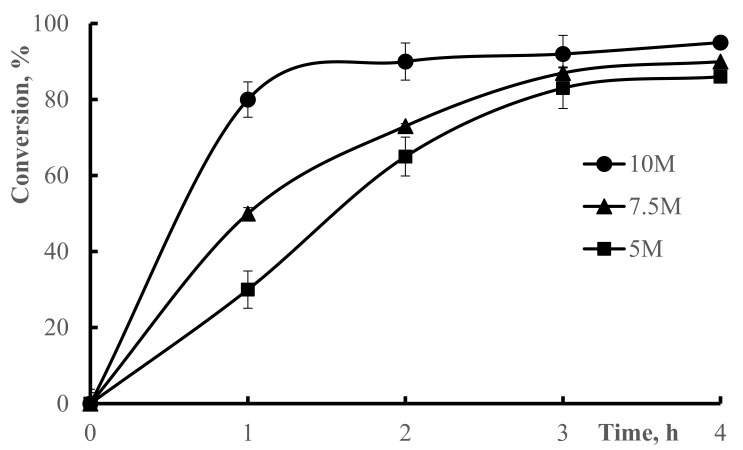
Conversion of 50–50% DMAA–AMPS hydrogel over time at various monomer concentrations. Conditions: initiator concentration 0.07% of total monomer mass, 0.74% crosslinker, reaction temperature 60 °C.

The initial concentration of monomer is very important for hydrogel conversion, because a large number of radicals determines the degree of polymerization [31]. Figure 1 indicates the conversion dependency on the reaction time at different monomer concentrations. 

As the concentration of monomer is high, the diffusion rate of the monomer increases, and a large number of active centers is produced, resulting in high conversion or polymerization in the end [32]. Moreover, with the increase of monomer concentration, the influence of monomer concentration on polymerization becomes significant.

As shown in Figure 1, conversion increases with increasing concentration of monomer. The greater the concentration of monomer, the greater the free-radical active centers form. The higher reactivity of monomer DMAA in the radical copolymerization reaction due to its resonance with a carboxyl group makes the monomers even more reactive in higher monomer concentrations [33]. When the monomer concentration reaches 10 M, a considerable increase is observed in the polymer conversion (*p* < 0.5), while at 7.5 M and 5 M the conversion of polymerization increased steadily (*p* < 0.5). It is clear from this figure that increasing monomer concentration caused increasing polymerization rate. In free-radical polymerizations, an auto-acceleration of polymerization rate is typically observed when the monomer concentration is high. 

The degree of polymerization was investigated at a constant monomer concentration as a change of temperature and initiator concentration. In the study of polymerization, the monomer concentration was fixed at 5 M (50–50% DMAA–AMPS molar ratio) and initiator concentration was 0.07% of the total monomer mass. The whole reaction time used was 4 h and was the same for all different temperatures. 

Conditions: monomer molar ratio DMAA–AMPS 50–50%, initiator concentration 0.07% of total monomer mass, 0.74% crosslinker, total monomer concentration 5M

**Figure 2:**
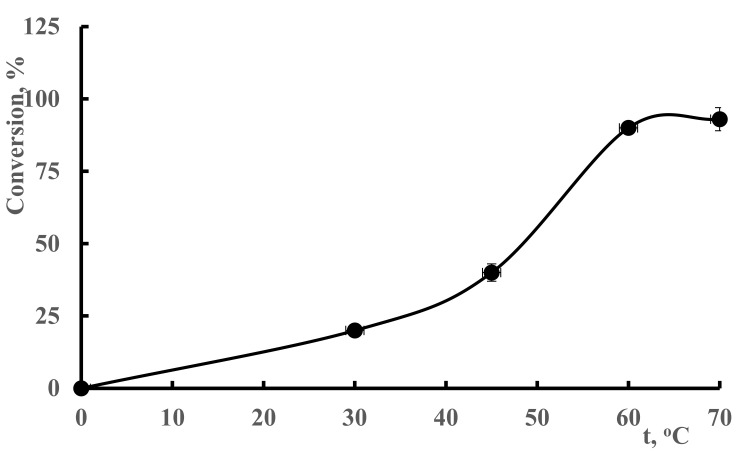
The influence of temperature on conversion of hydrogel synthesis.

The temperature effect on the conversion of polymerization is described in Figure 2. The radical concentration increases as the temperature rises, leading to an increase in the conversion of polymerization [34]. 

If the temperature is less than 30 °C, thermal decomposition of ammonium persulfate initiator will be decreased, which leads to the formation of a very little amount of active center that can polymerize. The conversion of polymerization increases as the temperature rises from 30–60 °C. This is because as the reaction conversion increases with temperature, the generation of free radicals decreases, resulting in a very low increase in polymer yield in the 60–65 °C temperature range. As high temperature causes an increase to the rates of chain termination reactions, a decrease was observed form 65 °C to 70 °C. 

It is clear from Figure 2 that the conversion of polymerization increases as temperature increases from 30 to 60 °C. However, after 65 °C, the conversion changes are not obvious. The increase of the conversion can be ascribed to two important factors. The first one is because of a higher reaction rate between the initiator and the backbone of the polymer. The second factor is because the diffusion of the initiator into the monomer increases with the increasing temperature. However, as can be seen from Figure 2, after 65 °C, the chain termination reaction occurs and thus forms small poymer chains [35]. 

**Figure 3:**
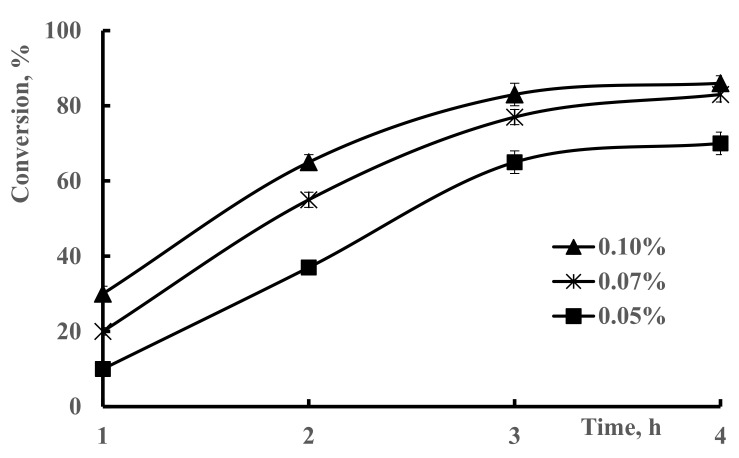
Influence of initiator concentration on conversion of 50–50% DMAA–AMPS hydrogel synthesis. Conditions: (0.74% crosslinker, total monomer concentration 5 M, reaction temperature 60 °C).

Figure 3 shows the relationship of conversion and polymerization time at different initiator concentrations. The conversion increases with the concentration of the initiator. The increase in the conversion of polymerization with increasing initiator concentration from 0.05% to 0.07% (*p* < 0.001) may be attributed to an increase in the active center of the monomer and initiator molecules in the polymerization medium, and an increase in the initiation and propagation rates of the polymer. However, further increase of the initiator (more than 0.1%) leads to a slight decrease in conversion. This can be explained by the higher rate of free-radical formation, leading to faster consumption of monomers. Accordingly, shorter chains are formed and easily washed out by solvents [36].

The swelling degree of the hydrogel was observed in distilled water at room temperature using the gravimetric method. The copolymers obtained at 5 M of monomer (50%–50% DMAA–AMPS molar ratio) and 0.07% of initiator concentration in total monomer mass were used to investigate their swelling at different pHs. 

**Figure 4:**
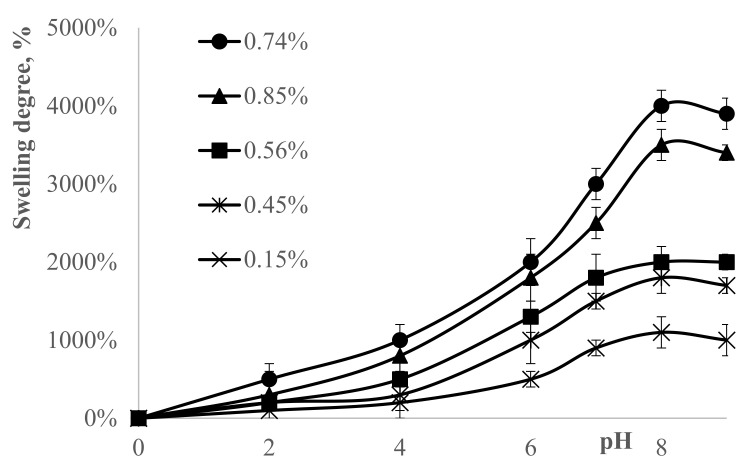
The pH effect on swelling degree at different concentrations of crosslinkers. Hydrogel (50–50% DMAA–AMPS molar ratio, initiator concentration 0.07% of total monomer mass, total monomer concentration 5 M, reaction temperature 60 °C) was tested.

A hydrogel consisting of DMAA and Na-AMPS should exhibit various swelling ratios at different pHs since it possesses two oppositely ionized functional groups and dissociates at that pH range. Figure 4 displays the swelling degree increases with increasing pH values. This is mainly due to the amine groups of the DMAA. The electrostatic repulsion among ionized groups causes the hydrogel to swell, which was relatively higher than DMAA–AAm hydrogel [37]. 

In the low-pH range, due to the protonation of the sulfonate anions, the hydrogen-bonding interaction between sulfonate groups dominates and leads to smaller hydrogels. Therefore, as the polymer network shrinks, water molecules are difficult to penetrate. As a result, swelling degrees of the hydrogels are reduced. On the contrary, at higher pH, sulfonate groups are ionized. Thus, electrostatic repulsion among sulfonate groups increases to swell the polymer networks [38,39]. However, with further increasing pH to 9, the “charge-screening effect” of the excess sodium ions in the swelling media shields the charge of sulfonate acid anions. Thus, electrostatic repulsion between polymer chains is decreased, resulting in a slight decrease in swelling degree [40,41]. The hydrogel networks increased with the increase of concentration of crosslinking agent, which resulted in adsorbing more water, and increasing the degree of swelling. For example, when the concentration of crosslinking agent was 0.74%, the hydrogel exhibits high swelling performance. More absorbed water (ions in the water) increases the electrostatic repulsion among sulfonate groups, which increases the swelling of the polymer networks. On the other hand, the less electrostatic repulsion occurs in the concentration of crosslinking agent 0.15% due to the less water inside the polymer networks. 

Statistical analyses were done for the above-mentioned different crosslinker concentrations. *p*-value for 0.15% was 0.00075, all the other concentration *p*-values were close to zero (*p* < 0.0001). Correlations with pH and swelling degree for all the crosslinker concentrations were higher than 0.95 (for 0.74% 0.968, for 0.85% 0.967, for 0.56% 0.973, for 0.45% 0.957, and for 0.15% 0.950). 

**Figure 5:**
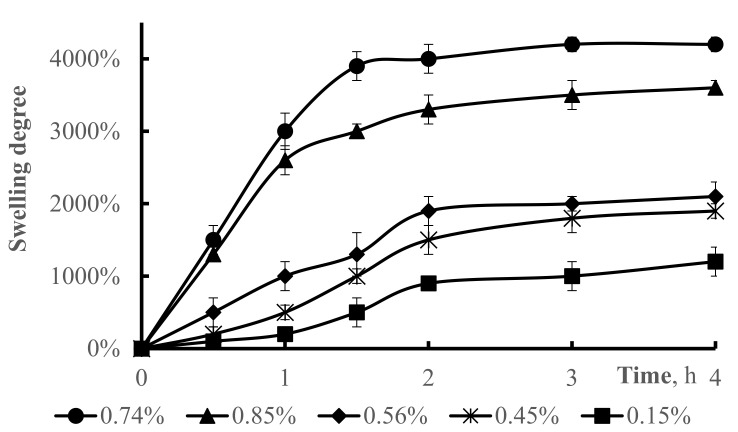
The effect of crosslinker concentration on the swelling degree of hydrogel. Hydrogel (50–50% DMAA–AMPS molar ratio, initiator concentration 0.07% of total monomer mass, total monomer concentration 5 M, reaction temperature 60 °C) was used.

Figure 5 shows the curves of swelling degree of the hydrogels. It can be seen from Figure 5 that the swelling degree increased as the concentration of crosslinker increased. When the crosslinker concentrations were 0.74% and 0.85% the swelling degree increased sharply over the first two hours (*p* < 0.001 for both concentrations). After two hours, *p*-values were 0.98 (for 0.74%) and 0.89 (for 0.85%) indicating that there were no significant changes. *p*-values for the bottom three crosslinker concentrations (0.56%, 0.45% and 0.15%) were 0.0070, 0.0011, and 0.0012, respectively. Generally, the swelling degree is influenced by the density of crosslinking of the synthesized hydrogel. On contrary, as for DMAA hydrogels, the hydrogel networks increased with the addition of crosslinking agent, which resulted in adsorbing more water, and increasing the degree of swelling. Thus, the swelling degrees increased with the increase of the concentration of crosslinking agent from 0.15% to 0.74%. However, with further increase of the crosslinking agent’s concentration, the swelling degree declines, which may suggest the greater degree of crosslinking of the hydrogel remarkably enlarges the friction among the polymeric chains and consequently reduces the space for water to enter in the process of swelling [42]. Thus, a lower swelling degree was observed.

### Metal-Ion Sorption Study

**Figure 6:**
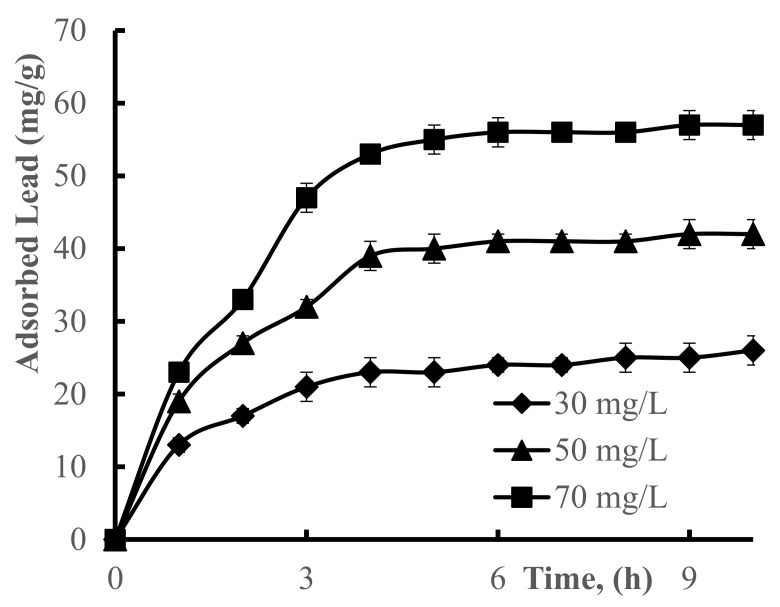
Pb(II) ion adsorption at different times, at various initial concentrations (30 mg/L, 50 mg/L, and 70 mg/L) of Pb(II) ions. Conditions: mass of dry hydrogel 100 mg, at 25 °C. Hydrogel (50–50% DMAA–AMPS molar ratio, initiator concentration 0.07% of total monomer mass, 0.74% crosslinker, total monomer concentration 5 M) was used.

Sorption of Pb(II) ions by crosslinked hydrogel was investigated based on the contact time and metal-ion concentration (30 mg/L, 50 mg/L and 70 mg/L) in solution. The results showed that adsorption of Pb(II) by the hydrogel was greatly dependent on the initial concentration of metal ions. Initially, after adding poly(DMAA–AMPS) hydrogel, sorption rate increases due to there being a lot of space in the hydrogel network, and an optimal removal efficiency was achieved in 4 h (Figure 6). When Pb(II) ion concentrations were 30 mg/L, 50 mg/L and 70 mg/L the sorption process increased for the first five hours (*p*-values 0.002, *p* < 0.001 and *p* < 0.001), and after 5 h there were no significant changes (*p* > 0.99 for all). Pearson’s correlation coefficients for all three values are (30 mg/L, 50 mg/L and 70 mg/L), which is higher than 0.9, suggesting a strong, positive association between three variables. 

**Figure 7:**
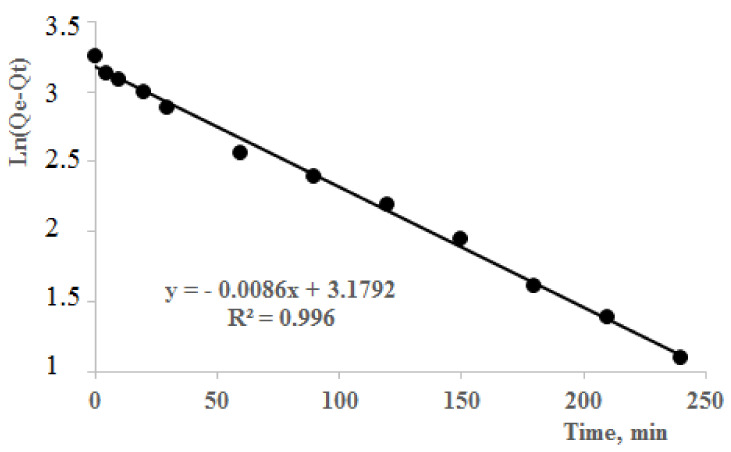
Pseudo-first-order models isotherms for Pb(II) removal by DMAA–AMPS hydrogel. Conditions: mass of dry hydrogel 100 mg; concentration of solution 30 mg/L and 25 °C. Hydrogel (50–50% DMAA–AMPS molar ratio, initiator concentration 0.07% of total monomer mass, 0.74% crosslinker, total monomer concentration 5 M) was used.

**Figure 8:**
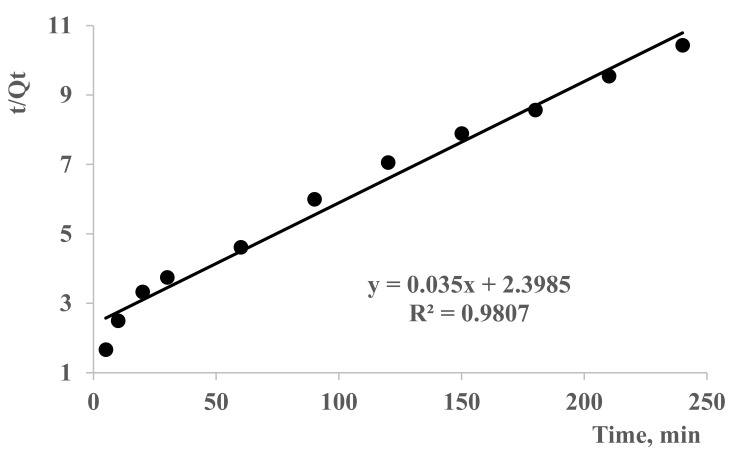
Pseudo-first-order models isotherms for Pb(II) removal by DMAA–AMPS hydrogel. Conditions: mass of dry hydrogel 100 mg; concentration solution of 30 mg/L and 25 °C. Hydrogel (50–50% DMAA–AMPS molar ratio, initiator concentration 0.07% of total monomer mass, 0.74% crosslinker, total monomer concentration 5 M) was used.

Figure 7 explains the pseudo-first-order model isotherms, while Figure 8 gives information on the pseudo-second-order isotherms. If the kinetic model best fits the pseudo-first-order reaction plot by giving R^2^ value close to 1, it indicates that the reaction is more inclined towards physisorption. Similarly, if the reaction fits well to pseudo-second-order model, it indicates an inclination towards chemisorption. Sorption kinetics of Pb(II) ions by poly(DMAA–AMPS) hydrogel was analyzed by pseudo-first-order and pseudo-second-order models (Figure 7 and Figure 8). Parameters for pseudo-first-order plots were used as follows: K_1_, Q_e_ were 0.0086, 24.03 mg/g and linearity was found to be 0.996. For pseudo-second-order models, parameters used were K_2_, Q_e_ and linearity were 0.003, 28.6 mg/g and 0.98, respectively.

Experimental value of Q_e_ was 26 mg/g (Figure 6). Both pseudo-first- and pseudo-second-order kinetics models gave a closer value of adsorption (Figure 7 and Figure 8). This shows that the pseudo-first and pseudo-second-order kinetics models both fit for adsorption kinetics [43,44,45,46,47]. However, Q_e_ with a lower experimental value was found in pseudo-second order, and vice versa in pseudo-first order. This phenomenon can be explained at early stages of the process by the diffusion effect due to greater Van Der Waals forces in the sorption mechanism. In addition, the chemisorption may be slow, and restrict the ion-exchange process during sorption [48,49].

## 4. Conclusions

In this study, poly[DMAA-co-AMPS] hydrogels were prepared via free-radical polymerization. The conversion hydrogels were studied in a different monomer, initiator, crosslinker concentration, and temperature.

In relatively higher monomer concentration (10 M), the resulting conversion was the highest. However, smaller concentration (7.5 M and 5 M) also reached nearly to the highest point. The optimum temperature for this hydrogel synthesis was found to be 60 °C. It was found that an initiator with high concentration increases the reaction rate, but does not always yield hydrogels. In terms of swelling, the swelling degree increased with the increase of pH. Finally, the optimum concentration of crosslinking agent for the best swelling of hydrogels was found to be 0.74% of total monomer weight.

After selecting the best-swelling hydrogel with high conversion, the metal adsorption capacity was tested. Based on our results, the optimal contact time for Pb(II) removal by DMAA–AMPS hydrogel was 5 h. Experimental equilibrium adsorption of Pb(II) ions (30 mg/L) 26 mg and pseudo-first- and pseudo-second-order kinetics models gave closer values of adsorption of 24 mg and 28 mg, respectively.

## Figures and Tables

**Table 1:** Initial calculation for standard errors.

Concentration	1 h Standard Errors	2 h Standard Errors	3 h Standard Errors	4 h Standard Errors
0.45%	0.65	0.54	0.50	0.39
0.85%	0.59	0.51	0.44	0.37

**Table 2:** Initial calculation for standard deviation and standard errors.

Concentration	1st Experiment	2nd Experiment	Standard Deviation	Root	Standard Error
0.45%	5	6.3	0.91	1.41	0.65

## Data Availability

The data presented in this study are available upon request from the corresponding author.
